# Time Trends in Trajectories of Forearm Mineral Content and Bone Size during Childhood—Results from Cross‐Sectional Measurements with the Same Apparatus Four Decades Apart

**DOI:** 10.1002/jbm4.10720

**Published:** 2023-02-03

**Authors:** Björn E. Rosengren, Jessica Karlsson, Erika Bergman, Henrik Ahlborg, Lars Jehpsson, Magnus K. Karlsson

**Affiliations:** ^1^ Clinical and Molecular Osteoporosis Research Unit, Department of Clinical Sciences and Orthopedics Lund University, Skåne University Hospital Malmö Sweden

**Keywords:** BONE MINERAL CONTENT, BONE MINERAL DENSITY, BONE, CHILDREN, CONTENT, DENSITY, MINERAL, PEDIATRIC, SIZE, STRENGTH, STRUCTURE, TIME TRENDS

## Abstract

Evidence suggests that single photon absorptiometry (SPA)‐measured forearm bone mineral density (BMD) is lower in contemporary children in Malmö than it was four decades ago, but the fracture incidence in the at‐risk population (all Malmö children) has been stable during the same period. The aim of this study was to evaluate if improvements in skeletal structure over time may explain this observation. In 2017–2018 we measured distal forearm bone mineral content (BMC; mg/cm) and periosteal diameter (mm) in 238 boys and 204 girls aged 7–15 using SPA. Based on the SPA measurements, we calculated forearm BMD (mg/cm^2^), bone mineral apparent density (BMAD, mg/cm^3^), section modulus, and strength index (BMAD × section modulus). The results were compared with those derived from measurements of 55 boys and 61 girls of the same ages using the same scanner in 1979–1981. We used log‐linear regression with age, sex, and cohort as predictors to investigate differences in trait trajectories (trait versus age slopes [mean percent difference in beta values (95% confidence interval)]). SPA‐measured forearm BMC was lower at each age in 2017–2018 compared to 1979–1981 (a mean age and sex adjusted relative difference of 9.1%), the forearm BMC trajectory was similar in 2017–2018 to that in 1979–1981 (reference) [0.0%/year (−1.0%, 1.0%)], while the 2017–2018 forearm periosteal diameter trajectory was steeper [1.1%/year (0.3%, 2.0%)]. Since bone size influences both BMD (BMC divided by scanned area) and mechanical characteristics, the forearm BMD trajectory was flatter in 2017–2018 [−1.1%/year (−2.0%, −0.2%)] and the forearm section modulus trajectory steeper [3.9%/year (1.4%, 6.4%)]. Forearm strength index trajectory was similar [1.8%/year (−0.5%, 4.1%)]. The lower SPA‐measured forearm BMD trajectory in contemporary children compared to four decades ago may be offset by changes in forearm bone structure, resulting in similar overall bone strength. © 2023 The Authors. *JBMR Plus* published by Wiley Periodicals LLC on behalf of American Society for Bone and Mineral Research.

## Graphical abstract

Distal forearm strength index (mean value of right and left forearm) was measured by single‐photon absorptiometry in two normative cohorts of boys and girls aged 7–15 in the periods 1979–1981 and 2017–2018. The strength index, which takes into account both the tissue mineral content and the structural appearance, was calculated as the product of the section modulus and the BMAD. Lines depict linear regression equations with 95% confidence interval bands from a log‐linear regression model (adjusted for age and sex).

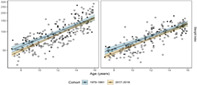

## Introduction

Thirty percent of children will suffer at least one fracture^(^ ^1^ ^)^ before age 18 and 50% of women and 22% of men will undergo any type of fracture after age 50.^(^ ^2^ ^)^ One of the greatest risk factors is low bone mineral density (BMD).^(^ ^3^, ^4^, ^5^ ^)^ However, BMD is just one characteristic of bone strength. Other material and structural properties, such as size, shape, cortical porosity, and three‐dimensional architecture, also contribute to bone strength.^(^ ^6^, ^7^, ^8^, ^9^ ^)^ The observed increase in bone fragility with aging is thus dependent on both loss of BMD^(^ ^6^ ^)^ and changes in bone structure.^(^ ^6^, ^9^ ^)^ This is clinically relevant because the risk of sustaining a fragility fracture is independently associated with BMD,^(^ ^3^ ^)^ bone size,^(^ ^10^ ^)^ and bone structure.^(^ ^9^ ^)^

One determinant of BMD is physical activity,^(^ ^11^, ^12^ ^)^ which has a more significant effect on BMD in weight‐loaded than in non‐weight‐loaded regions.^(^ ^13^ ^)^ However, studies using both single photon absorptiometry (SPA)^(^ ^14^ ^)^ and dual X‐ray absorptiometry (DXA)^(^ ^15^ ^)^ have shown that BMD in such unloaded skeletal regions as the distal forearm are also higher in physically active than in inactive individuals, although with less magnitude in the benefit in BMD than in weight‐loaded regions. Studies have further shown that both scanning techniques (SPA and DXA) predict fracture similarly.^(^ ^3^, ^4^, ^5^ ^)^

However, the level of physical activity has decreased,^(^ ^16^, ^17^ ^)^ and BMD in the general population is also lower than it previously was.^(^ ^18^, ^19^, ^20^ ^)^ This was demonstrated by DXA in weight‐loaded skeletal regions in US adults and US elderly in 2014 compared to 2005,^(^ ^18^, ^19^ ^)^ as well as by SPA in forearms in Swedish children in 2017–2018 compared to 1979–1981.^(^ ^20^ ^)^ In fact, when estimating forearm BMD, BMD in 16‐year‐old children was estimated to be 1 SD lower than the mean in 2017–2018 compared to 40 years earlier.^(^ ^20^ ^)^ Such a difference ought to result in a doubled fracture incidence.^(^ ^4^, ^5^, ^21^ ^)^ Yet studies in the same population, where the time trend in forearm BMD was shown,^(^ ^20^ ^)^ were unable to confirm any change in fracture occurrence between 1979 and 2018.^(^ ^22^, ^23^ ^)^ This raises the question of whether the lower forearm BMD^(^ ^20^ ^)^ has been offset by structural changes that preserve the bone's resistance to fractures. Similar ideas have been suggested in the elderly, where research has shown that the age‐dependent decline in forearm BMD in Swedish women is associated with an increase in bone size.^(^ ^6^, ^7^, ^8^ ^)^ Similar findings were also reported from the Third National Health and Nutrition Examination Survey (NHANES III), that reduction in DXA‐measured BMD in the hip does not necessarily mean reduced mechanical strength.^(^ ^24^ ^)^

Both body height and weight are associated with BMD as well as bone structure.^(^ ^25^ ^)^ The proportion of Swedish children aged 16–19 years who are overweight and obese doubled from 1980 to 2017,^(^ ^26^ ^)^ and the young adult body height in Sweden increased by a mean of 2 cm from 1979 to 2018.^(^ ^26^ ^)^ Therefore, it seems reasonable to postulate that these time trends may have influenced BMD and bone structure.

We posed the following research questions. First, is the recently reported downturn in childhood forearm BMD^(^ ^20^ ^)^ associated with larger forearm periosteal apposition, which would counteract the lower tissue mineral content, so that a strength index, which accounts for both the tissue mineral content and the structural appearance of the bone, is similar today to what it was four decades ago? Second, can any weight and height differences explain forearm BMD and/or any forearm bone structure differences? As the pediatric fracture incidence from 1979 to 2018 was stable in the at‐risk population (all Malmö children),^(^ ^20^, ^22^, ^23^ ^)^ we hypothesized that children in the years 2017–2018 developed a larger bone size than children in the years 1979–1981, at least partly as a consequence of being taller and heavier.

## Methods

### Study participants 1979–1981

Malmö is a city in southern Sweden whose population was 235,111 (38,651 < 16 years) in 1979.^(^ ^27^ ^)^ The participants were 116 children (55 boys and 61 girls) aged 7–15 years, all with Caucasian ethnicity. They were living in the city in 1979–1981 and participated in a non‐population‐based study that collected normative anthropometry and bone mass data.^(^ ^28^ ^)^ The children in this study were volunteers who were asked to participate, predominantly the children or acquaintances of employees in our department. There was no general invitation in newspapers or flyers. Furthermore, we have no specific information on the number that were included from each group or the number of invited children who declined participation and no lifestyle data on participants.

### Study participants in 2017–2018

In 2017 the city population was 333,633 (64,309 < 16 years).^(^ ^27^ ^)^ A total of 976 children (491 boys and 485 girls) aged 7–15 years were invited to participate in a study in 2017–2018 to collect normative anthropometry and bone mass data.^(^ ^20^ ^)^ All children in three publicly funded primary schools, allocated according to their residential address, were invited to participate. The children were invited through written flyers in the schools, and both students and parents/guardians had to sign an agreement on participation. In all, 442 children (238 boys and 204 girls) accepted participation (45%). Of these, 95% were of Caucasian ethnicity.

### Measurements of anthropometry

We used standard equipment in both 1979–1981 and 2017–2018 to measure weight (kilograms) and height (centimeters).

### Measurements of bone tissue mineral

**Fig. 1:**
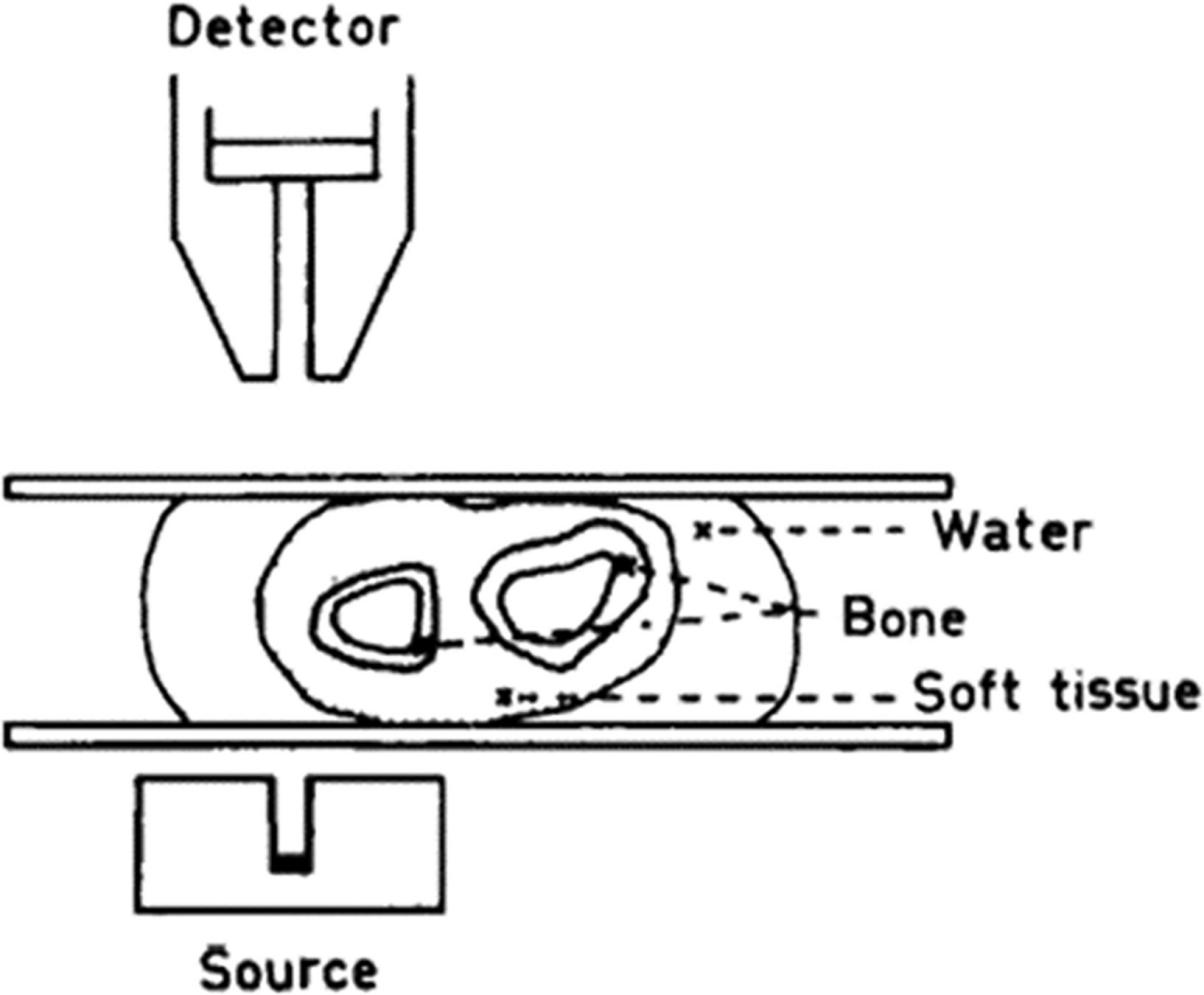
The single‐photon absorptiometry (SPA) equipment as it was depicted in the original description.^(^ ^6^, ^29^ ^)^ All individuals had a rubber water cuff around the forearm so as to obtain the same baseline value. The SPA method measures absorption over a line, in our case 6 cm proximal to the styloid process of the ulna, from the medial to the lateral side of the distal forearm. During the scan, the radiation source and the collimator were moved 1 mm at each step between the measuring points.

**Fig. 2:**
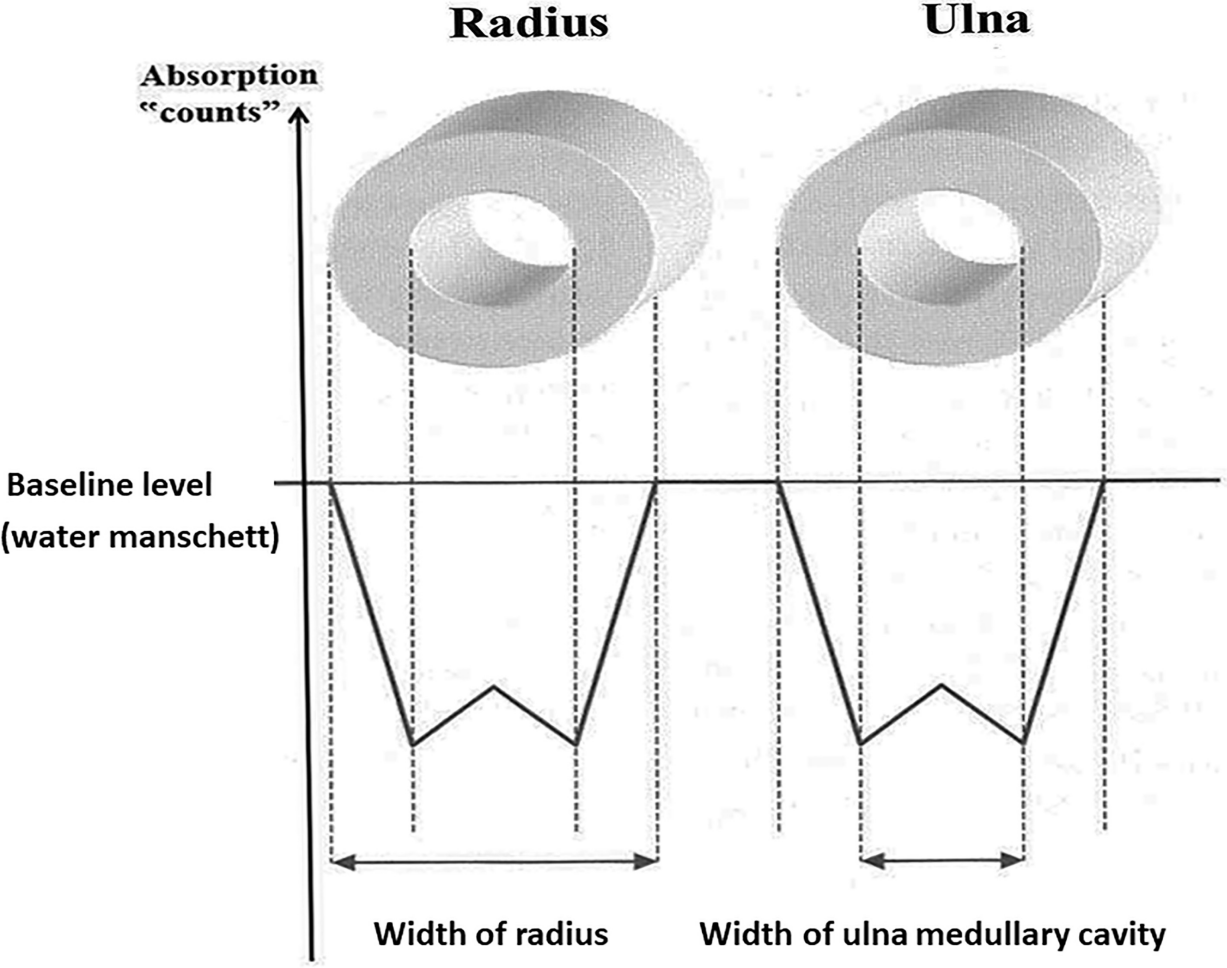
Trace of output from single‐photon absorptiometry equipment. When plotting all measured points, we achieved an integral that was proportional to the amount of mineral within the scanned axle. When transferring the BMC (g/cm) value to BMD (g/cm^2^), we divided the estimated amount of mineral within the scanned distance by the width of the bones and an added standardized height of 1 cm.

We used SPA in the forearm 6 cm proximal to the styloid process of the ulna to measure bone mineral content (BMC in milligrams per centimeter [mg/cm] of bone length). The technique uses a rectilinear scan across the radius and ulna, with a radiation source (241 Americium) and a detector moving simultaneously, according to the method of Nauclér et al.^(^ ^29^ ^)^ All individuals had a rubber water cuff around the forearm so they would receive the same baseline value (Fig. 1). We scanned both the right and the left arm. Since radius and ulna at this site are similar in bone width, it is not possible at this level, based on the graphs, to identify which of the bones is radius and which is ulna (Fig. 2). That is, we were not able to take individual measurements for each bone. Instead, we used the averages of the four bones in our calculations. For participants with an incident fracture in the upper extremity during the preceding year, we only measured the uninjured arm. We excluded scans with technical measurement errors. Only four children measured in 1979–1981 and nine children measured in 2017–2018 had an upper extremity fracture during the previous year, and five unilateral scans in children measured in 2017–2018 had technical measurement errors. By these criteria, we used measurements from only one arm for four children (3.4%) measured in 1979–1981 (two nondominant arms and two with unknown dominance) and 14 children (3.2%) measured in 2017–2018 (seven nondominant arms and seven dominant arms). We calculated forearm BMD (in milligrams per square centimeter) as BMC divided by the estimated scanned area and bone mineral apparent density (BMAD, in milligrams per cubic centimeter [mg/cm^3^]) as BMC divided by the estimated cortical area.

### Measurements of bone structural characteristics

We measured the forearm periosteal diameter, the forearm medullary diameter, and the forearm cortical thickness of the distal radius from the graph of the scans, as previously described in detail^(^ ^6^ ^)^ (Fig. 2). All measurements were validated and analyzed in random order by one researcher. The total skeletal cross‐sectional area, the forearm medullary area, and the forearm cortical area were calculated on the assumption that the bone is cylindrical (area = diameter^2^ × π/4).^(^ ^6^ ^)^ A key geometric variable, called the cross‐sectional moment of inertia, was calculated according to the formula ([periosteal diameter/2]^4^ – [medullary diameter/2]^4^) × π/4.^(^ ^6^ ^)^ Another variable, called the section modulus, an estimate of the ability of a circular structure to withstand bending forces, was calculated as the cross‐sectional moment of inertia divided by half the periosteal diameter.^(^ ^6^, ^30^ ^)^ Previous cadaver studies verified that the cross‐sectional moment of inertia in this region is highly correlated with the strength of the distal radius.^(^ ^31^ ^)^ An estimate of the volumetric tissue mineral content, expressed as the BMAD (mg/cm^3^), was calculated as the BMC divided by the cortical area.^(^ ^6^ ^)^ The strength index, which takes into account both the tissue mineral content and the structural appearance, was calculated as the product of the section modulus and the BMAD.^(^ ^6^, ^30^ ^)^ The strength index was previously shown to correlate well with mechanical strength in long bones of rats.^(^ ^32^ ^)^

The same densitometer was used to measure study participants in 1979–1981 and 2017–2018. There was no long‐term drift in BMD between 1979–1981 and 2017–2018, as evaluated by a standardized phantom (mean 0.1% per year [95% confidence interval, CI: −0.2, 0.4]), or in bone width (0.057 mm per year [95% CI: −0.324, 0.439]). The bone strength parameters were calculated from the bone width measures and the coefficient of variation were at the same magnitude. The phantom included two 2‐cm‐wide cylinders, placed in an aqueous solution that included potassium phosphate (K_2_PO_4_) covered by plastic. The BMD value in the phantom was 1.25 g/cm^2^. This should be compared to a mean BMD value of 0.33 g/cm^2^ in our children. The radiation source was replaced in 1980. Phantom measurements were then used to adjust the measured values.^(^ ^6^ ^)^ In this study, we divided the average phantom value during the period 2017–2018 with the average phantom value during the period 1979–1981, divided in measurements done before and after the radiation source was changed. All values in the period 1979–1981 were then multiplied by one of these constants, depending on whether the measurement was done before (where the constant was 1.002) or after (where the constant was 1.034) the radiation source was changed. If we had not used the correction coefficients, we found a difference in BMD of 3.4% when comparing our two cohorts, depending only on drift in the technical equipment (drift in the radiation source and/or drift in the detection of the radiation in the detector). The precision (coefficient of variation [CV]) of the SPA measurements in the forearm was 2.7% for BMD when determined by 311 standardized phantom measurements and 4.8% when determined by three repeated measurements of 20 different arms (after repositioning). The CV for bone width was 8.0%. All precision measurements were done in adults. One technician performed all measurements in 1979–1981 while two technicians did the measurements in 2017–2018. All measurements followed the standard protocol for distal forearm bone scanning.^(^ ^6^ ^)^

### Statistics

We used R version 4.0 and R Studio version 1.3 for all statistical calculations. Since the 1979–1981 cohort was already measured when this study was planned, the power calculations were, to a large extent, regulated according to the 1979–1981 sample size. To reach a higher power, we invited all children in all classes in three schools to the later measurement, rendering more than three times as many study participants in 2017–2018 as in 1979–1981. To investigate the associations between the outcome measurements and cohort, we used log‐linear regression. We used two different model specifications, one adjusted for only age and sex (Model 1), the other adjusted for age, sex, height, and weight (Model 2). When modeling height adjusted for age and sex, we also added a squared‐age term, including interaction with sex, to account for the remaining nonlinear pattern in the log‐transformed growth data. In both models, we included interaction terms between age and sex, as well as between age and cohort. The model fit is presented separately for boys and girls in figures as scatter plots overlaid with fitted slopes and 95% confidence bands to describe uncertainty. We regarded a *p*‐value of *p* < 0.05 as a statistically significant difference. The Ethical Review Board in Lund, Sweden, approved the study (Reference No. 2016/1680). Written consent was obtained from participants and parents/guardians of each participant before study start.

## Results

**Fig. 3:**
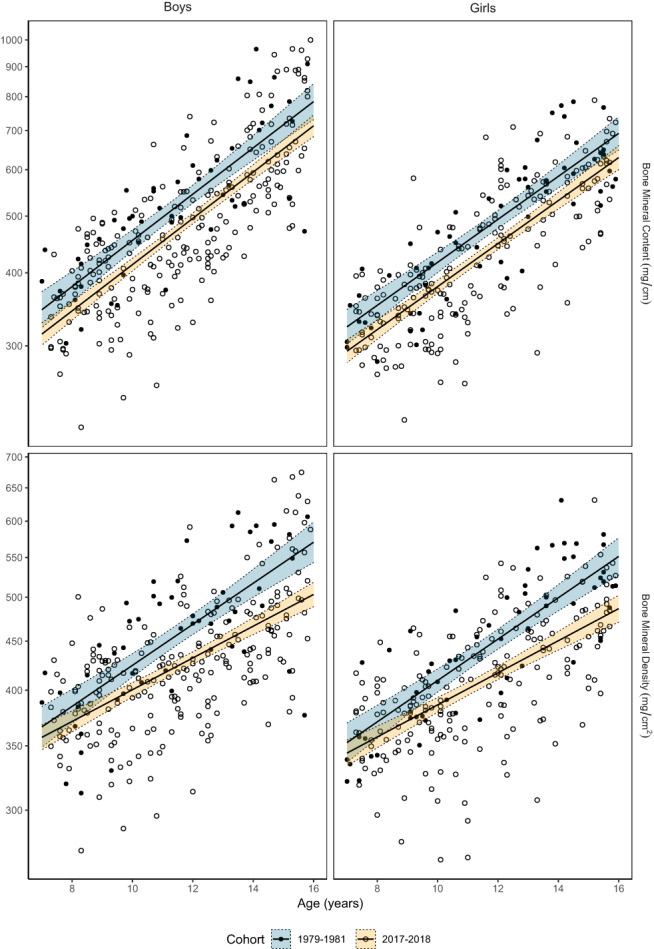
Forearm bone mineral content (BMC) and bone mineral density (BMD) (mean value of right and left forearm) measured using single‐photon absorptiometry in normative cohorts of boys and girls in 1979–1981 and in 2017–2018. Scatter plots overlaid with fitted slopes and 95% confidence bands from log‐linear regression model (model 1; adjusted for age and sex).

The forearm BMC trajectory was similar for children measured in 2017–2018 and 1979–1981 (reference cohort) [mean difference beta value 0.0%/year (−1.0%, 1.0%)]. The 9.1% lower intercept values at age 7 (Table 1), together with the similar inclination of the slopes (Table 1 and Fig. 3), indicate that the 2017–2018 cohort at each age had a lower forearm BMC value compared to the 1979–1981 cohort.

**Table 1:** Model Estimates from Regression Analyses on Cortical Site Traits of Distal Radius 6 cm Proximal to Styloid Process of Ulna

	Outcome measures (dependent variables)										
		Bone mineral content (mg/cm)	Bone mineral density (mg/cm^2^)	Bone mineral apparent density (mg/cm^3^)	Periosteal diameter (mm)	Medullary diameter (mm)	Cortical thickness (mm)	Total cortical area (cm^2^)	Cross‐sectional moment of inertia	Section modulus	Strength index
Predictors (Independent Variables)	Intercept	**345.930** *** **(322.028, 371.607)**	**366.327** *** **(348.755, 384.785)**	**590.672** *** **(559.989, 623.036)**	**9.455** *** **(9.067, 9.860)**	**4.025** *** **(3.682, 4.400)**	**2.804** *** **(2.654, 2.962)**	**0.704** *** **(0.647, 0.765)**	**0.042** *** **(0.035, 0.050)**	**0.087** *** **(0.076, 0.098)**	**51.106** *** **(45.347, 57.596)**
	Cohort (2017–2018)	**0.909** ** **(0.846, 0.977)**	0.975 (0.928, 1.025)	**1.084** ** **(1.027, 1.145)**	**0.931** *** **(0.893, 0.971)**	0.970 (0.885, 1.063)	**0.889** *** **(0.841, 0.941)**	**0.867** *** **(0.797, 0.943)**	**0.710** *** **(0.595, 0.846)**	**0.772** *** **(0.678, 0.880)**	**0.838** ** **(0.741, 0.947)**
	Age * cohort (2017–2018)	1.000 (0.986, 1.014)	**0.989** * **(0.980, 0.998)**	**0.980** *** **(0.970, 0.990)**	**1.011** ** **(1.003, 1.020)**	1.016 (0.999, 1.033)	1.008 (0.998, 1.019)	1**.023** ** **(1.007, 1.039)**	**1.052** ** **(1.019, 1.087)**	**1.039** ** **(1.014, 1.064)**	1.018 (0.995, 1.041)
	Sex	**0.935** * **(0.881, 0.992)**	0.963 (0.925, 1.003)	0.978 (0.935, 1.023)	0.971 (0.938, 1.005)	0.985 (0.914, 1.061)	0.979 (0.935, 1.025)	0.942 (0.879, 1.009)	0.937 (0.811, 1.081)	0.951 (0.855, 1.059)	0.930 (0.842, 1.029)
	Age	**1.095** *** **(1.080, 1.110)**	**1.050** *** **(1.041, 1.060)**	**1.022** *** **(1.012, 1.032)**	**1.042** *** **(1.034, 1.051)**	**1.061** *** **(1.043, 1.078)**	**1.022** *** **(1.011, 1.032)**	**1.087** *** **(1.069, 1.104)**	**1.162** *** **(1.125, 1.199)**	**1.118** *** **(1.092, 1.145)**	**1.142** *** **(1.117, 1.168)**
	Age * Sex	0.994 (0.982, 1.005)	1.000 (0.993, 1.008)	1.006 (0.998, 1.015)	0.993 (0.987, 1.000)	0.989 (0.975, 1.003)	0.996 (0.987, 1.004)	0.987 (0.974, 1.000)	**0.969** * **(0.943, 0.995)**	**0.977** * **(0.957, 0.997)**	0.983 (0.965, 1.002)

*Note*: Two normative cohorts of children aged 7–15 years were measured using single‐photon absorptiometry. The first cohort included 55 boys and 61 girls measured in 1979–1981 and the second 238 boys and 204 girls measured in 2017–2018. When evaluating the cohort, we used the 1979–1981 cohort as a reference, and when evaluating sex, we used the boys as a reference. To investigate associations between the outcome measures (dependent variables) in the 2017–2018 cohort in relation to the 1979–1981 cohort (reference), we used log‐linear regression, adjusted for age (each year from age 7) and sex, with interaction terms between age and sex, and age and cohort (Model 1). When modeling height adjusted for age and sex, we also added a squared‐age term, including interaction with sex, to account for the remaining nonlinear pattern in the log‐transformed growth data. The variable *intercept* should be interpreted as the absolute values in 7‐year‐old boys in the 1979–1981 cohort. The variable *cohort* should be interpreted as the relative differences at age 7 when comparing the 2017–2018 cohort with the 1979–1981 cohort, and the variable age * cohort should be interpreted as the relative differences in the annual change when comparing the 2017–2018 cohort with the 1979–1981 cohort. The remaining values in the model are presented for completeness without being further discussed in the paper. Data are shown as antilogs of beta value with the 95% confidence interval within brackets. Statistically significant differences are bolded.

* *p* < 0.05.

** *p* < 0.01.

*** *p* < 0.001.

**Fig. 4:**
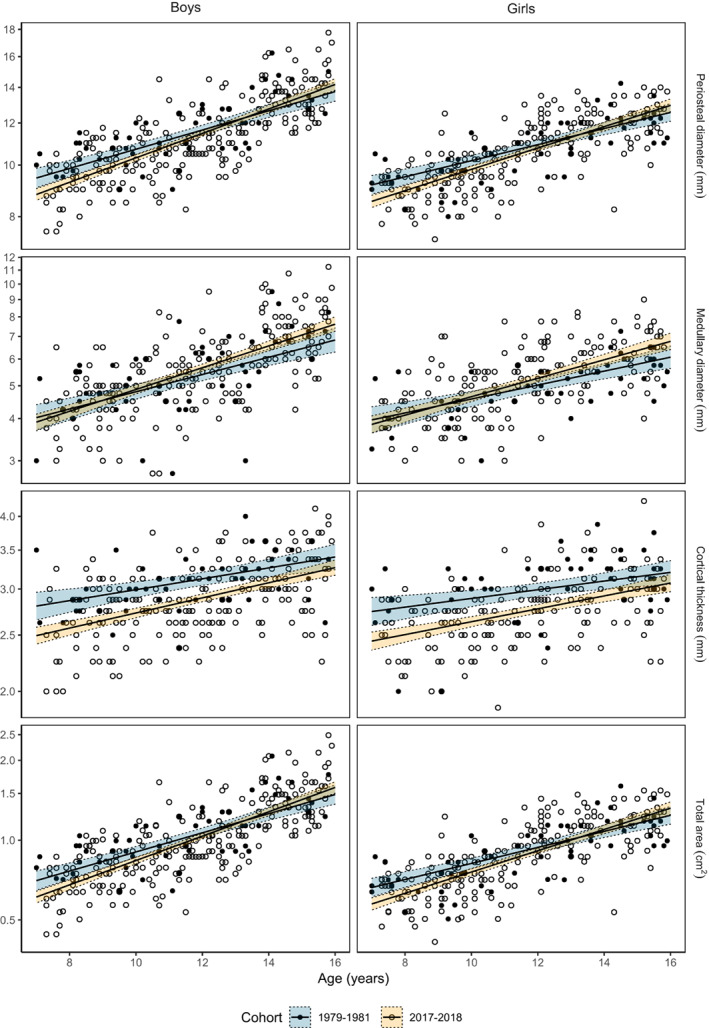
Forearm bone structure (mean value of right and left forearms) measured using single‐photon absorptiometry in normative cohorts of boys and girls in 1979–1981 and in 2017–2018. Scatter plots overlaid with fitted slopes and 95% confidence bands from log‐linear regression model (model 1, adjusted for age and sex).

The forearm periosteal diameter trajectory was steeper in children measured in 2017–2018 compared to 1979–1981 [1.1%/year (0.3%, 2.0%)]; the forearm medullary diameter trajectory may have been steeper, but differences did not reach statistical significance [1.6%/year (−0.1%, 3.3%)] (Table 1, Fig. 4). The forearm cortical thickness trajectories were similar in the 2017–2018 and 1979–1981 cohorts [0.8%/year (−0.2%, 1.9%)] (Table 1, Fig. 2).

**Fig. 5:**
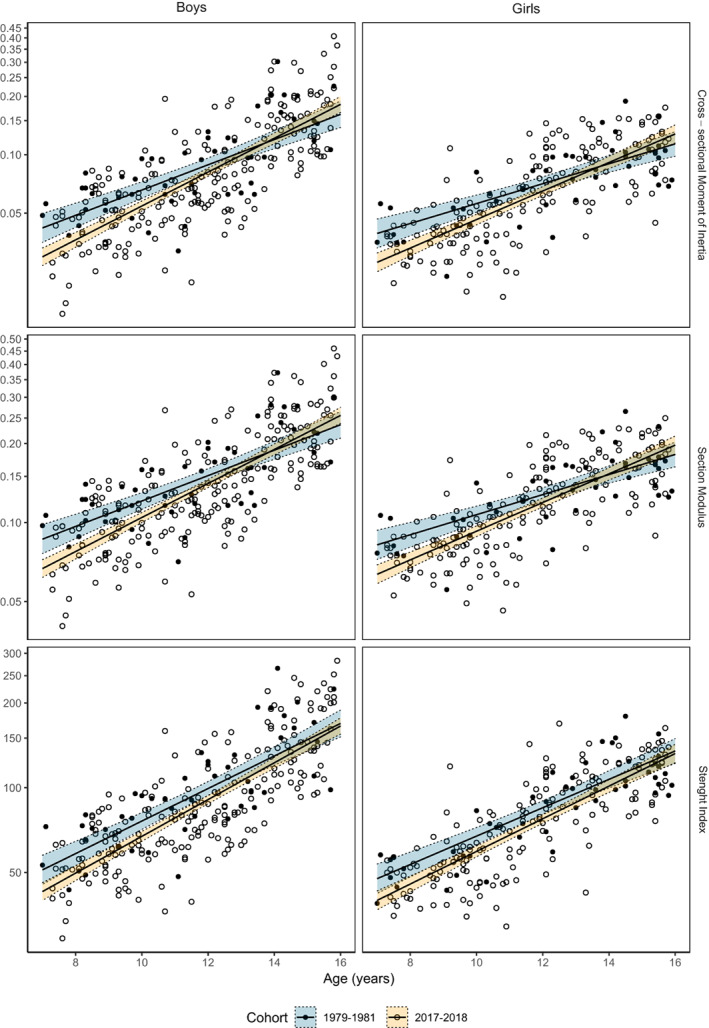
Strength calculations in distal forearm (mean value of right and left forearms) measured using single‐photon absorptiometry in normative cohorts of boys and girls in 1979–1981 and in 2017–2018. Scatter plots overlaid with fitted slopes and 95% confidence bands from log‐linear regression model (model 1, adjusted for age and sex).

The forearm cross‐sectional moment of inertia trajectory [5.2%/year (1.9%, 8.7%)] and the forearm section modulus age slope [3.9%/year (1.4%, 6.4%)] were steeper in children measured in 2017–2018 compared to 1979–1981 (Table 1, Fig. 5). The forearm strength index trajectory (taking both BMAD and section modulus into account) was similar in children measured in 2017–2018 and 1979–1981 [1.8%/year (−0.5%, 4.1%)] (Table 1, Fig. 5).

All results remained after adjustment for height and weight (Table 2).

**Table 2:** Model Estimates from Regression Analyses on Cortical Site Traits of the Distal Radius 6 cm Proximal to the Styloid Process of the Ulna

	Periosteal diameter (mm)	Medullary diameter (mm)	Cortical thickness (mm)	Total cortical area (cm^2^)	Bone mineral content (mg/cm)	Bone mineral density (mg/cm^2^)	Bone mineral apparent density (mg/cm^3^)	Cross‐sectional moment of inertia	Section modulus	Strength index
Intercept	**4.784** *** **(4.088 5.598)**	**1.156** *** **(0.827 1.614)**	**2.350** *** **(1.860 2.969)**	**0.180** *** **(0.132 0.247)**	**158.911** *** **(120.017 210.410)**	**331.83** ** **(269.430 408.682)**	**758.409** *** **(605.336 950.189)**	**0.003** *** **(0.002 0.006)**	**0.012** *** **(0.008 0.020)**	**9.425** *** **(6.125 14.502)**
Sex (female)	**0.953** ** **(0.925 0.983)**	0.943 (0.882 1.007)	0.976 (0.931 1.022)	***0.908*** ** **(0.855 0.965)**	***0.911*** *** **(0.863 0.961)**	***0.955*** * **(0.918 0.994)**	0.977 (0.934 1.021)	**0.858** * **(0.757 0.973)**	**0.892** * **(0.813 0.980)**	**0.872** ** **(0.801 0.949)**
Age (years from age 7)	0.999 (0.989 1.009)	0.982 (0.963 1.002)	**1.015** * **(1.001 1.029**)	0.998 (0.979 1.017)	**1.033** *** **(1.016 1.051)**	**1.034** *** **(1.021 1.047)**	**1.024** *** **(1.010 1.037)**	0.995 (0.958 1.032)	0.997 (0.970 1.025)	1.021 (0.995 1.047)
Cohort (2017–2018)	**0.931** *** **(0.897 0.966)**	0.973 (0.898 1.054)	**0.890** *** **(0.841 0.941)**	**0.867** *** **(0.805 0.934)**	**0.910** ** **(0.852 0.972)**	0.977 (0.930 1.026)	**1.083** ** **(1.026 1.144)**	**0.714** *** **(0.613 0.832)**	**0.776** *** **(0.693 0.870)**	**0.841** ** **(0.758 0.933)**
Sex * Age	1.001 (0.995 1.007)	1.004 (0.991 1.016)	0.997 (0.988 1.006)	1.002 (0.990 1.014)	1.004 (0.994 1.015)	1.004 (0.996 1.011)	1.006 (0.998 1.015)	0.998 (0.975 1.022)	0.999 (0.981 1.017)	1.005 (0.989 1.022)
Age * Cohort	**1.009** * **(1.002 1.016)**	1.010 (0.996 1.025)	1.008 (0.998 1.018)	**1.018** * **(1.004 1.032)**	0.996 (0.984 1.009)	**0.987** ** **(0.978 0.997)**	**0.980** *** **(0.970 0.989)**	**1.042** ** **(1.013 1.071)**	**1.031** ** **(1.010 1.052)**	1.010 (0.991 1.029)

*Note*: Two normative cohorts of children aged 7–15 years were measured using single‐photon absorptiometry. The first cohort included 55 boys and 61 girls measured in 1979–1981 and the second 238 boys and 204 girls measured in 2017–2018. When evaluating the cohort, we used the 1979–1981 cohort as reference, and when evaluating sex, the boys were used as reference. To investigate associations between the outcome measurements and cohort in this model (model 2) we used log‐linear regression adjusted for age, sex, height, and weight with interaction terms between age and sex as well as age and cohort (Model 2). When modeling height adjusted for age and sex, we also added a squared‐age term, including interaction with sex, to account for the remaining nonlinear pattern in the log‐transformed growth data. The variable *intercept* should be interpreted as the absolute values in 7‐year‐old boys in the 1979–1981 cohort. The variable *cohort* should be interpreted as the relative differences at age 7 when comparing the 2017–2018 cohort with the 1979–1981 cohort, and the variable age * cohort should be interpreted as the relative differences in the annual change when comparing the 2017–2018 cohort with the 1979–1981 cohort. The remaining values in the model are presented for completeness without being further discussed in the paper. Data are shown as antilogs of beta value with a 95% confidence interval within brackets. Statistically significant differences are bolded.

* *p* < 0.05.

** *p* < 0.01.

*** *p* < 0.001.

**Fig. 6:**
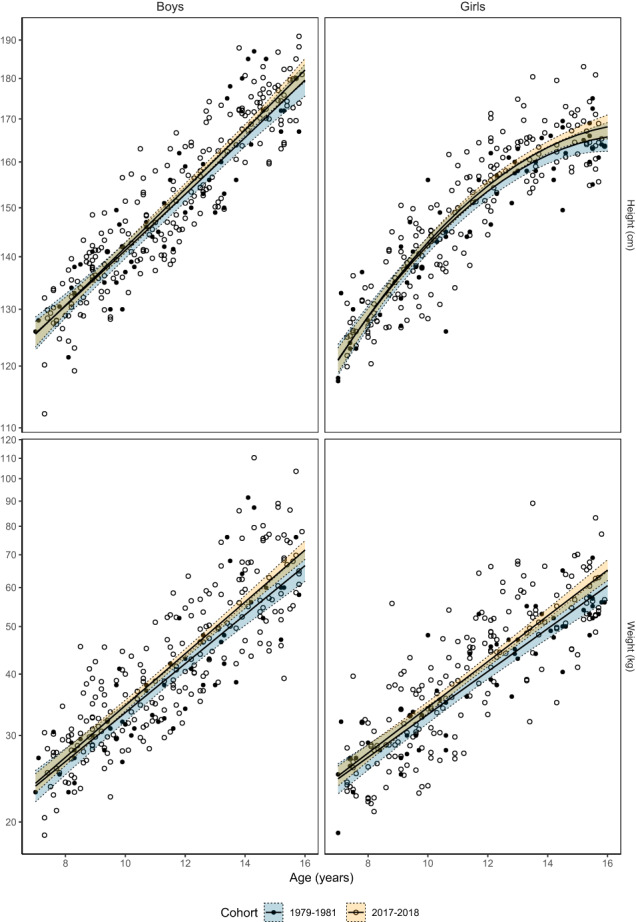
Anthropometric data measured using standard equipment in normative cohorts of boys and girls in 1979–1981 and in 2017–2018 in relation to age. Scatter plots overlaid with fitted slopes and 95% confidence bands from log‐linear regression model (model 1, adjusted for age and sex).

We used the age‐ and sex‐adjusted log‐linear regression slopes to predict trait values at age 16 (Fig. 6). Boys and girls in the 2017–2018 cohorts at age 16 were taller (boys +2.5 cm [1.4%], girls +2.3 cm [1.4%]) and heavier (boys +5.1 kg [7.6%], girls+4.6 kg [7.6%]) compared to their counterparts in 1979–1981. The predicted forearm BMC value was also lower at this age (boys −71.2 mg/cm [9.1%], girls −62.2 mg/cm [9.1%]), forearm periosteal width larger (boys +0.43 mm [3.1%], girls +0.39 mm [3.1%]), forearm cross‐sectional moment of inertia higher (boys +0.02 [12.5%], girls +0.01 [12.4%]), forearm section modulus higher (boys +0.02 [8.6%], girls +0.02 [8.5%]) and forearm strength index lower (boys −3.5 [2.0%], girls −2.8 [2.1%]).

Twenty‐three percent of the children measured in 1979–1981 and 19% of the children measured in 2017–2018 reported a previous fracture (*p* = 0.31).

## Discussion

We recently reported that the pediatric forearm BMD at age 16 in Malmö, Sweden, was estimated to be 1.0 SD lower today than four decades ago.^(^ ^20^ ^)^ According to the literature, this difference ought to translate into a doubled fracture incidence.^(^ ^4^, ^5^, ^21^ ^)^ However, we have not been able to verify this when evaluating children in the at‐risk population (all Malmö children).^(^ ^22^, ^23^ ^)^ As BMD (in milligrams per square centimeter) is based on a two‐dimensional imaging technique,^(^ ^4^, ^6^, ^29^ ^)^ problems will arise when estimating bone strength using BMD in growing skeletons and skeletons with different sizes. This is because a lower two‐dimensionally estimated BMD could be the result of (i) a lower amount of tissue mineral content within an anatomic region with a fixed size, (ii) a larger bone size in a region with a fixed amount of tissue mineral content, or (iii) a combination of a lower amount of tissue mineral content and larger bone size. Lower tissue mineral content will lead to a weaker skeleton, whereas a larger bone size will lead to a stronger skeleton.^(^ ^6^, ^7^, ^8^, ^30^, ^32^ ^)^ Therefore, a lower BMD resulting solely from a larger bone size will give the false impression of a weaker bone, something that would hypothetically explain the stable fracture incidence in the at‐risk population.^(^ ^22^, ^23^ ^)^

The fact that we found similar fracture incidences throughout the 1979–2018 period in the pediatric population from which the measured cohorts originated^(^ ^22^, ^23^ ^)^ raises the question of whether there had been time trends in bone traits that counteracted the lower forearm BMD.^(^ ^20^ ^)^ One way that would enable bone strength to be retained despite a lower tissue mineral content could be to have the cortical shell farther from the long axis of the bone since the resistance of bone to bending and torsional forces is increased by the fourth power of the radius of the bone.^(^ ^6^, ^7^, ^30^, ^32^ ^)^ The current study supports this hypothesis when finding greater inclination in the periosteal apposition age trajectory in children in 2017–2018 than in children in 1979–1981, which results in a favorable development of the mechanical characteristics of the bone. The finding of a greater periosteal apposition is also of clinical relevance because bone size predicts fractures independently of BMD.^(^ ^10^ ^)^ Finally, when taking into account both time trends in tissue mineral content and periosteal apposition in the calculated strength index, we found a similar strength index trajectory in the two cohorts. This is in line with previous reports of stable pediatric fracture incidence in the at‐risk population during the evaluated period.^(^ ^22^, ^23^ ^)^

When discussing our data in relation to fracture incidence during the evaluated period, we must emphasize that, even though BMD and bone structure are important for fracture risk, other factors that contribute to fracture risk may have changed, such as risk‐taking behavior, fracture protective devices, structured traffic, and home environment safety work, as well as exposure to trauma.^(^ ^5^, ^21^, ^22^, ^23^ ^)^ There may also have been time trends in structural characteristics not measured in this study that are known to be important for bone strength. These include intracortical porosity, trabecular connectivity, and other bone micro‐architectural traits, as well as bone turnover, all of which influence fracture risk but cannot be evaluated with SPA.

Children nowadays are generally taller and weigh more than four decades ago,^(^ ^27^ ^)^ and both body height and weight are associated with BMD and bone structure.^(^ ^25^ ^)^ That is, taller and heavier individuals in general have longer and wider bones. However, all our inferences remained after adjustment for group differences in height and weight. This indicates that the group differences in this study could not be attributed to differences in body anthropometry. Therefore, we speculate that children in the 2017–2018 period may have developed bone with less tissue mineral content than children in the 1979–1981 period and then adapted by developing a greater bone width with the aim of preserving bone strength, or that children nowadays develop wider bones that result in less mechanical stress on the bone tissue and, thus, less bone mineral accrual. However, other factors, such as secular changes in hormonal status, pubertal age, patterns of physical activity, sedentary behavior, nutritional intake, calcium intake, and/or intake of soft drinks, may be factors contributing to time trends in skeletal development.

The study's strengths include the use of the same scanner, phantom measurements that excluded long‐term drift in the apparatus during the studied period, data that allowed us to adjust for the changed radiation source, that all scans were plotted by a single researcher in random order, and that all children were from the same city. Study limitations include the cross‐sectional study design that only allowed us to estimate but not prospectively measure bone trait changes during growth. The small sample size and the nonrandom recruitment in 1979–1981 are other concerns that introduced the risk of selection bias. Differences in bone size may have provided differences in the partial volume effect. However, we speculate that, because the difference in bone width was minimal in absolute values, this would be a minor problem. Moreover, precision measurements for bone width might have been inferior in children. Bone width measurement depends on bone edge detection, and generally this detection has a higher precision in denser bones. That is, it may be more difficult, based on graphs, to estimate the borders in small bones and bones with a lag in mineralization. Another possible flaw in the study is that the mean BMD of the phantom was four times higher than the mean BMD of the study sample. It is therefore possible that the adjustment factor we used did not accurately represent the drift in the machine at BMD levels that were one fourth the BMD values of the phantom.

Another concern is that we do not have data on forearm bone length. Boys and girls in the period 2017–2018 had greater body height than boys and girls in the 1979–1981 period. If there was also a difference in forearm bone length, it is possible that the children in 2017–2018 were measured proportionately closer to the distal end of the forearm than children in 1979–1981. We tried to address this issue by adjusting in model 2 for body height, but this is only a proxy for forearm bone length. Much of the late adolescent growth in height is due to growth in the spine, not long bones. However, girls measured in 2017–2018 had a mean forearm bone length that was 1.4% higher and boys had a mean forearm bone length that was 1.1% higher than girls and boys, respectively, at the same ages in 1979–1981,^(^ ^20^ ^)^ and such a small difference at the 6‐cm region would be a minor problem since the width of the forearm bone at this level is quite constant. Furthermore, if a difference in measured distance from the wrist between the cohorts had been a confounding factor, this would have affected our intercept values but not our trajectories, the basis of all our conclusions in this paper. Since most children were of Caucasian ethnicity and lived in socioeconomically middle‐class areas, it is questionable whether the results can be transferred to children from other ethnic backgrounds and/or living in other socioeconomic areas. Further limitations include a lack of information on lifestyle and health that could provide clues as to the reasons for the different patterns of BMD and bone structure development in the cohorts. It might also have been advantageous to have bone measurements taken using the present gold standard for evaluation of BMD, DXA, as well as with three‐dimensional techniques, since both techniques have higher precision for BMD measurements than the SPA method. With a higher precision it would have been possible to identify even smaller differences as being statistically significant. With, for example, peripheral quantitative computed tomography (pQCT), it would have been possible to estimate geometrical measurements at both the ultradistal radius and the distal radius, as well as both cortical and trabecular regions. However, DXA and pQCT were not available in 1979–1981, and studies have further shown that SPA estimates distal forearm BMD with a strong correlation to DXA measurements and that both scanning techniques predict fractures similarly.^(^ ^3^, ^4^, ^5^ ^)^ Another advantage would have been to measure different anatomical regions and use high‐resolution pQCT so as to be able to also evaluate group differences in bone microstructure.

The lower forearm BMD age trajectory in Malmö children today compared with four decades ago seems partly to be the result of a steeper forearm width age trajectory that counteracts a decrease in strength index. Results do not seem related to group differences in height or weight. Due to the small sample size, our results ought to be verified in larger studies, preferably including modern scanning techniques.

## Author Contributions

**Bjorn Erik Rosengren:** Conceptualization; data curation; investigation; methodology; supervision; validation; writing – original draft. **Jessica Karlsson:** Data curation; project administration; validation; writing – original draft. **Erika Bergman:** Data curation; investigation; methodology; project administration; resources; supervision; validation; writing – original draft. **Henrik G Ahlborg:** Data curation; investigation; methodology; supervision; validation; visualization; writing – original draft. **Lars Jehpsson:** Data curation; formal analysis; investigation; methodology; project administration; software; validation. **Magnus K Karlsson:** Conceptualization; data curation; formal analysis; funding acquisition; investigation; methodology; supervision; visualization; writing – original draft.

## Conflict of Interest

None of the authors have any conflict of interest. Funders had no influence on study design, data collection, data analysis, interpretation of data, writing of the report, or decision on where to submit the paper.

### Peer Review

The peer review history for this article is available at https://publons.com/publon/10.1002/jbm4.10720.

## Data Availability

The data are not publicly available due to privacy and ethical restrictions. Extended data that support the findings of this study are available on request from the corresponding author.
